# Propolis–carboxymethyl cellulose Janus nanoparticles as multifunctional bioactive additives in stirred yogurt

**DOI:** 10.1038/s41598-026-48430-5

**Published:** 2026-04-13

**Authors:** Roghayieh Razavi, Rahim Molaei, Amin Alipashaeihalabi, Ahmad Mirani, Negar Alizadeh, Mohammad Abbaszadeh, Mehran Moradi

**Affiliations:** 1 Department of Food Hygiene and Quality Control, Faculty of Veterinary Medicine, Urmia University, 1177, Urmia, Iran; 2 Materials Synthesis Laboratory, Carbon Tech Industrial Group, Carbon Tech, Urmia, Iran

**Keywords:** Janus particles, Stirred yogurt, Propolis, Functional food, Nanomaterials, Biochemistry, Biotechnology, Microbiology, Nanoscience and technology

## Abstract

This study developed innovative Janus nanoparticles (JPs) using propolis and carboxymethyl cellulose (CMC) to enhance the technological and functional properties of stirred yogurt. The synthesized propolis-CMC JPs, with an average particle size of 13.40 nm ± 3.56 μm, exhibited remarkable antioxidant activity in both DPPH and FRAP assays, along with potent antibacterial effects against common foodborne pathogens, including *Escherichia coli* O157:H7 and *Listeria monocytogenes*, with minimum inhibitory concentrations of 1.5–2.0 mg/mL. Yogurt samples fortified with 0.5, 1, and 5% propolis-CMC JPs were evaluated during 21 days of storage. Incorporation of propolis-CMC JPs, particularly at 5%, significantly reduced syneresis, improved water-holding capacity, and inhibited microbial growth (3.55 ± 0.2 vs. 4.95 ± 0.3 log₁₀ CFU/g in control). Propolis-CMC JPs -enriched yogurts also showed enhanced antioxidant potential and favorable rheological characteristics. Sensory analysis revealed that yogurt with 1% propolis-CMC JPs achieved the highest color and aroma scores, while the 5% formulation exhibited the best texture attributes. These findings confirm that propolis-CMC JPs can act as natural emulsifiers, antioxidants, and antimicrobial agents, effectively addressing key technological limitations of yogurt production.

## Introduction

Yogurt is a widely consumed fermented dairy product produced by lactic acid fermentation of milk. Among various yogurt types, stirred yogurt is industrially prepared by completing fermentation in large tanks before packaging, resulting in a smooth and homogeneous texture^1^. In addition to being a rich source of proteins, carbohydrates, fats, vitamins, and minerals^2^, yogurt confers numerous health benefits, including the support of digestion, alleviation of lactose intolerance, enhancement of immunity, and protection against infections and colon cancer^3^. Despite its nutritional and functional advantages, yogurt quality is often compromised by microbial contamination, structural defects, and physicochemical instability issues. Improper milk handling, temperature fluctuations, and weak packaging can reduce the shelf life and safety of milk^4^. One of the most common structural challenges is syneresis, which arises from the rearrangement of casein micelles within the gel network, leading to phase separation and undesirable texture^5^. Gel stability is influenced by multiple factors, including pH, calcium concentration, fat content, total solid content, and storage conditions. Consequently, maintaining the structural integrity of yogurt during storage remains a key technological challenge.

To address these limitations, natural stabilizers and bioactive compounds have been increasingly incorporated into yogurt formulations. Hydrocolloids, polysaccharides, gums, and plant-derived extracts have been explored to enhance the texture, water retention, antioxidant activity, and microbial stability of meat products^6,7^. To address these concerns, recent research has focused on nanotechnology, particularly Janus nanoparticles (JPs), which offer multifunctional properties. JPs are colloidal particles with asymmetric surfaces that offer unique opportunities owing to their multifunctional properties, including emulsification, controlled release of bioactives, and antimicrobial activity^8,9^. The amphiphilic nature of JPs allows them to act as both hydrophilic and hydrophobic modifiers, making them ideal candidates for stabilizing multiphasic systems such as emulsions and dairy gels^10,11^. Although JPs have demonstrated potential in various fields, their application in real food systems, particularly in dairy products, remains limited^9^.

Consumer demand for natural preservatives has increased, prompting interest in plant extracts, essential oils, and apicultural products to improve food quality and safety^12^. Propolis, a natural resinous substance produced by honeybees, is rich in polyphenols, flavonoids, and bioactive compounds and exhibits well-documented antioxidant, antimicrobial, anti-inflammatory, and immunomodulatory activities. It is widely used in the food and pharmaceutical sectors owing to its natural source, affordability, and significant bioactive effects^13,14^. Propolis exhibits thermoplastic behavior, becoming malleable and adhesive when heated, but turning rigid and fragile upon cooling, particularly at sub-zero temperatures. This brittleness persists even when the temperature is increased^15^. However, its direct use in aqueous food systems is limited because of poor solubility and dispersion.

One of the important applications of JPs in food systems is their use in stabilizing water-in-oil or oil-in-water emulsions and serving as carriers of bioactive compounds in functional foods. This functionality arises from their characteristics as molecular surfactants and colloidal Pickering particles^9^. Naturally derived JPs have been proposed as potential and promising alternatives for stabilizing thermodynamically unstable food-grade Pickering emulsions^16^. It has been demonstrated that JPs can enhance the stability of Pickering emulsions by optimizing the water–oil interfacial area and the ratio of hydrophilic to hydrophobic domains^16^. This study presents the first application of propolis-based JPs as multifunctional natural nanostructures to improve the technological, microbiological, and sensory stabilities of stirred yogurt. The current study integrates amphiphilic structure JPs to achieve dual functionality within a single system, enhanced interfacial stabilization and bioactive delivery. Hybrid JPs from CMC as a hydrophilic component and propolis as a hydrophobic and bioactive phase. This nature not only addresses the poor solubility and limited dispersion of propolis in aqueous dairy matrices but also allows for controlled interactions with milk proteins, leading to improved gel integrity, water retention, and reduced syneresis. Although JPs have shown promising performance in biomedical, pharmaceutical, and packaging applications, their direct incorporation into food systems, particularly fermented dairy products, remains largely unexplored. Therefore, this study aimed to develop, characterize, and apply propolis-CMC JPs as a novel multifunctional additive in stirred yogurt and evaluate their effects on the physicochemical, microbial, and sensory properties of yogurt during storage.

## Materials and methods

### Materials

*Escherichia coli* O157:H7 (PTCC 1399) and *Listeria monocytogenes* (PTCC 1298) were obtained from the Iranian Research Organization for Science and Technology (IROST, Tehran, Iran). Propolis was supplied by BehdashtGhostar (Urmia, Iran). Culture media including Plate Count Agar (PCA) (Ibresco, Tehran, Iran), Mueller Hinton Agar (MHA) (Ibresco), and Tryptic Soy Broth (TSB) (Quelab, Montreal, Canada) were used for microbial investigation. DPPH (2,2-diphenyl-1-picrylhydrazyl) (Sigma-Aldrich, Madrid, Spain), deionized water (Diofaw, Urmia, Iran), and methanol (Pars Alcohol, Shiraz, Iran) were used for antioxidant evaluation. CMC (degree of substitution 0.9, molecular weight: 1.05058, high dynamic viscosity :2% water 25 °C, 50–150 mPa. s) and other chemicals and reagents were purchased from Merck (Darmstadt, Germany), unless specified otherwise.

### Propolis-CMC JPs synthesis

For the preparation of propolis extract, the method described by Chang et al.^17^, was followed with significant modifications to adapt the method to the propolis-CMC system. Briefly, 5 g of raw propolis was ground and dissolved in 100 mL of chloroform and stirred for 2 h. The mixture was centrifuged (Farzaneh Arman Co., Isfahan, Iran) at 10,000 rpm for 10 min, and the supernatant was collected. The second centrifugation removed the wax and other insoluble residues, then solvent evaporation at 40 °C to obtain propolis powder. Propolis-CMC JPs were synthesized based on the self-assembly approach as described by Razavi et al.^18^. One hundred milliliters of 0.5% CMC solution (in deionized water, pH 6.9) and propolis were prepared at a ratio of 1:2 (w/w). The resulting mixture was stirred uniformly at 650 rpm for 48 h at 25 °C.

### Characterization of propolis-CMC JPs

#### Instrumental characterization

The particle size distribution of the propolis-CMC JPs was measured using a scatteroscope particle size analyzer (PSA, Qudix Inc., South Korea). The morphology was examined using Field Emission Scanning Electron Microscopy (FESEM; SIGMA VP-500; Zeiss, Germany). The working distance for each image was 30 kV and recorded at 5 and 10kx magnifications. Crystalline properties were assessed by X-ray diffraction (XRD; Shimadzu, Tokyo, Japan), patterns were recorded over a 2θ range of 5°–80° using a Philips X-ray diffractometer equipped with a Cu-Kα1–2 radiation source (λ = 1.54 Å) operating at 45 kV and 40 mA. Chemical structures were characterized using Fourier Transform Infrared Spectroscopy (FTIR, Thermo Electron, Nexus® 670, Madison, USA) over 450–4000 cm⁻^1^ with 32 scans at a resolution of 4 cm⁻^1^.

#### Antimicrobial and antioxidant activities

The antimicrobial effects of propolis-CMC JPs were assessed using agar well diffusion. Bacterial suspensions of *E. coli* O157:H7 and *L. monocytogenes* (10⁸ CFU/mL) were swabbed onto MHA plates. Wells with a diameter of 6 mm were loaded with 100 μL of different propolis-CMC JPs concentrations (0.25, 0.5, 1, 5, and 10%) dissolved in deionized water. Plates were incubated at 37 °C for 24 h, and inhibition zones were measured in millimeters^19^. Deionized water was used as the solvent control. Minimum inhibitory concentration (MIC) and minimum bactericidal concentration (MBC) were determined using broth dilution in 96-well plates. Each well received 160 μL TSB, followed by propolis-CMC JPs at final concentrations of 0.5–4 mg/mL. Subsequently, 20 μL of bacterial suspension (10⁶ CFU/mL) was added. Plates were incubated at 37 °C for 24 h. MIC was defined as a 2 log₁₀ CFU/mL decrease (no visible growth) in bacterial count, while MBC corresponded to a 3 log₁₀ CFU/mL reduction (≥ 99.9%), both confirmed by colony counting^20^.

Antioxidant activity of CMC, propolis, and propolis-CMC JPs was evaluated using DPPH and FRAP assays. For the DPPH assay, 1 mL of DPPH solution (2 mg/50 mL pure methanol) was added to 1 mL of each material concentration (0.25, 0.5, 1, 2, 5, and 10%) and was kept for 60 min at 25 °C in darkness. The absorbance was then recorded at 517 nm with a UV–visible spectrophotometer, and the percentage of radical scavenging (% RS) was obtained according to Eq. 1^21^.

1 $$\% {\mathrm{RS}} = \left\{ {{{\left( {{\mathrm{A}}_{{{\mathrm{blank}}}} - {\mathrm{A}}_{{{\mathrm{sample}}}} } \right)} \mathord{\left/ {\vphantom {{\left( {{\mathrm{A}}_{{{\mathrm{blank}}}} - {\mathrm{A}}_{{{\mathrm{sample}}}} } \right)} {{\mathrm{A}}_{{{\mathrm{blank}}}} }}} \right. \kern-0pt} {{\mathrm{A}}_{{{\mathrm{blank}}}} }}} \right\} \times {1}00$$

where A_blank_ and A_sample_ represent the absorption rate of the DPPH solution and the DPPH solution containing each material, respectively.

For the FRAP assay, the FRAP reagent was freshly prepared by mixing 0.3 M acetate buffer (pH 3.6), 20 mM ferric chloride hexahydrate (FeCl₃·6H₂O) solution, and 10 mM TPTZ (2,4,6-tripyridyl-s-triazine) dissolved in 40 mM HCl, at a ratio of 10:1:1 (v/v/v). An aliquot of 150 µL of each material was then mixed with 2850 µL of the FRAP reagent and incubated in the dark at 23 ± 1 °C for 30 min. Following incubation, the absorbance of the reaction mixture was measured at 593 nm using a uv–vis spectrophotometer^18^.

### Yogurt preparation

Stirred yogurt was prepared as described by Gonçalves et al.^22^ with modifications. Fresh whole cow’s milk was standardized to 3.0% (w/w) fat and 12% (w/w) total solids. The milk was then homogenized and subjected to heat treatment at 95 °C for 5 min. After heat treatment, the milk was cooled to 42.5 ± 0.5 °C. The starter culture consisted of *Streptococcus salivarius* subsp*. thermophilus* and *Lactobacillus delbrueckii* subsp. *bulgaricus.* The inoculated milk was incubated at 42 °C until the pH reached 4.6. After fermentation, the propolis-CMC JPs were incorporated at concentrations of 0.5%, 1%, 5% (w/w). These concentrations were selected based on preliminary investigations to determine the minimum amount providing the maximum beneficial effect. Each concentration was mixed separately into the yogurt. Samples were transferred into capped containers and stored at 4 °C. Physicochemical, microbial, antioxidant, and sensory analyses, including pH, total titratable acidity (TTA), viscosity, water-holding capacity (WHC), syneresis, texture profile analysis (TPA), color, and overall acceptability, were conducted weekly over a 21-day storage period. A control without Propolis-CMC JPs was prepared using the same procedure as described above.

### Physicochemical properties of yogurt

#### pH and total titratable acidity

The pH of the yogurt was measured using a digital pH meter (Metrohm, Herisau, Switzerland). TTA was determined according to the AOAC (2005).

#### Water holding capacity and syneresis

Forty grams of yogurt was centrifuged at 7000 × *g* for 15 min at 4 °C (Farzaneh Arman Co., Isfahan, Iran). WHC (%) was calculated (Eq. 2).

2 $${\text{WHC }}\left( \% \right) \, = \, \left[ {{{\left( {{\mathrm{W}}_{{0}} {-}{\text{ W}}_{p} } \right)} \mathord{\left/ {\vphantom {{\left( {{\mathrm{W}}_{{0}} {-}{\text{ W}}_{p} } \right)} {{\mathrm{W}}_{0} }}} \right. \kern-0pt} {{\mathrm{W}}_{0} }}} \right] \times {1}00$$

where W₀ is the initial sample weight and Wₚ is the pellet weight^23^.

Syneresis was determined by centrifuging 40 g of yogurt at 3000 × *g* for 15 min ^24^. The separated whey was weighed, and syneresis (%) was calculated as follows (Eq. 3):

3 $${\mathrm{Syneresis}}\left( \% \right) = \left( {{{{\mathrm{Separated}}\;{\mathrm{liquid}}\;{\mathrm{weight}}} \mathord{\left/ {\vphantom {{{\mathrm{Separated}}\;{\mathrm{liquid}}\;{\mathrm{weight}}} {{\mathrm{Yogurt}}\;{\mathrm{weight}}}}} \right. \kern-0pt} {{\mathrm{Yogurt}}\;{\mathrm{weight}}}}} \right) \times {1}00$$

#### Viscosity

Yogurt viscosity was measured at 4 °C using a Rapid Visco Analyzer (RVA-TecMaster, Perten, Sweden) with 50 mL samples. A No. 5 rotor was used at 100 rpm for 30 s.

#### Texture profile analysis

Texture properties were measured using a texture analyzer (Stable Micro Systems, Surrey, UK) with a 5 kg load cell and 40 mm cylindrical probe. A compressive force was applied over 30 mm at a speed of 1 mm/s for 5 s. Parameters included firmness, adhesiveness, springiness, cohesiveness, gumminess, and chewiness. The samples were equilibrated at 20 ± 1 °C before testing^25^.

#### Antioxidant activity

Yogurt extracts were prepared by mixing 10 g of the sample with 50 mL of methanol–water (8:2 v/v), centrifuged at 5800 × *g* for 10 min, and filtered through Whatman No.1 paper, as described by Pham et al.^26^. DPPH radical scavenging activity was assessed by mixing 250 μL extract with 3 mL of 60 mM DPPH in 95% methanol, incubated in the dark for 1 h at 25 °C ^27^. Absorbance at 517 nm was recorded, and radical scavenging (%) was calculated as follows (Eq. 4):

4 $${\mathrm{Radical}}\;{\text{scavenging }}\left( {{\% }} \right) = \frac{{{\mathrm{A}}\;{\mathrm{control}} - {\mathrm{A}}\;{\mathrm{sample}}}}{{{\mathrm{A}}\;{\text{control }}}} \times { }100$$

where A _control_ and A _sample_ represent the absorption rates of the DPPH solution and the DPPH solution containing yogurt water extract, respectively.

#### Microbial analysis

Total bacterial count (TBC) was determined by homogenizing 10 g yogurt in 90 mL 0.1% peptone water, preparing serial dilutions, and plating on PCA via the pour plate method. Plates were incubated at 37 °C for 72 h ^1^.

#### Sensory and color analysis

Color (L*, a*, and b* values) was measured in triplicate using a Lovibond LC 100 spectrocolorimeter, and the total color difference (ΔE) was recorded. Sensory evaluation was performed using a 9-point hedonic scale (1 = Dislike Extremely, 5 = Neutral, and 9 = Like Extremely) for odor, color, texture, and overall acceptance. Ten semi-trained panelists (7 men, 3 women) assessed coded samples individually (without eating or tasting) in separate booths under incandescent light at 25 °C and 65% relative humidity^18^. All procedures involving human participants were carried out in accordance with the relevant institutional and international ethical guidelines and regulations. The sensory evaluation in this study was limited to visual inspection and general appearance assessment of yogurt samples, and no tasting or consumption of food products occurred. All experimental protocols were approved by Urmia University Ethics Committee. All participants were fully informed about the nature of the evaluation and voluntarily provided informed consent prior to participation.

### Statistical analysis

All experiments were performed at least three times. Data were analyzed using ANOVA in GraphPad Prism version 8.02 (GraphPad Software Inc., San Diego, CA, USA). Significant differences among means were determined by Tukey’s multiple-range test at *p* < 0.05.

## Results and discussion

### Characterization of propolis-CMC JPs

**Fig. 1:**
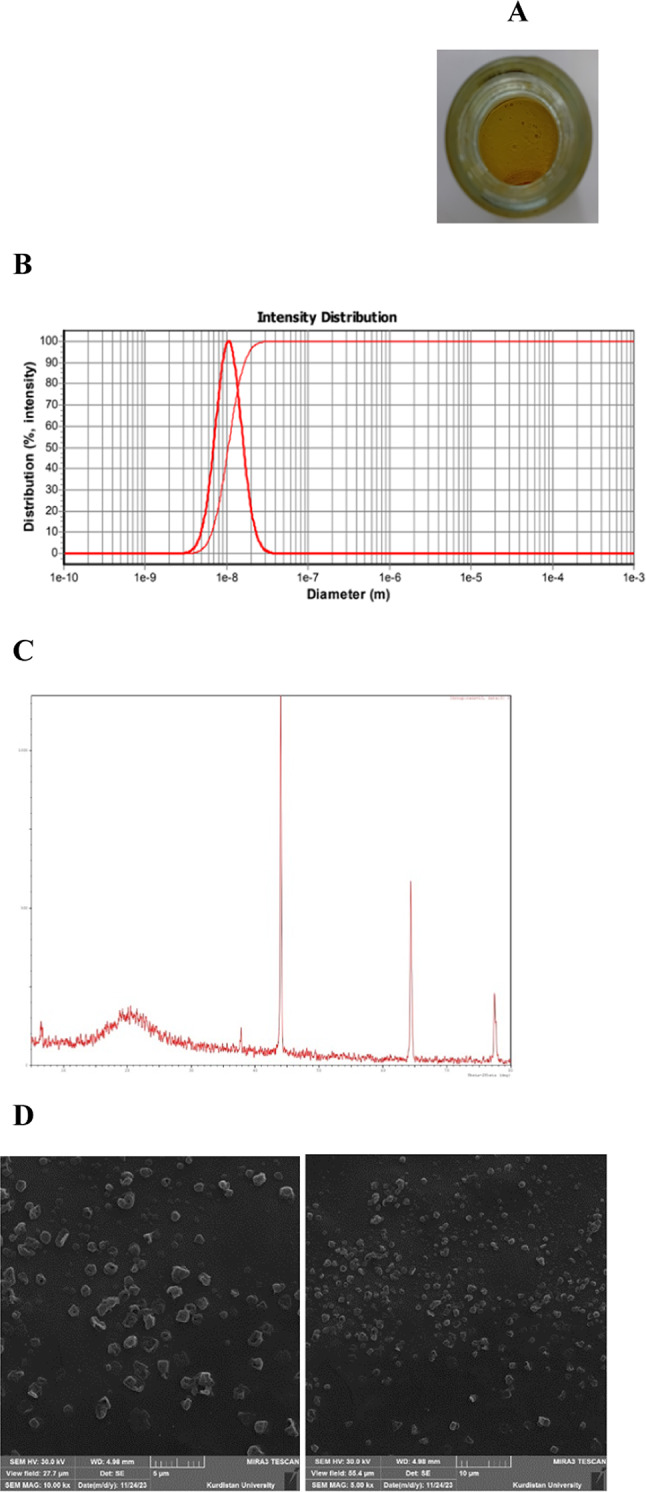
The image of propolis-CMC Janus nanoparticles (JPs) colloidal sample (**A**) and instrumental characterization of JPs: (**B**) particle size distribution by Scatter scope PSA, (**C**) crystallinity by XRD, and (**D**) morphology by FESEM.

Figure 1A shows an image of the propolis-CMC JPs colloidal solution. PSA revealed that over 99% of the particles were smaller than 37.7 nm ± 13.2, with an average diameter of 13.40 nm ± 3.56 (Fig. 1B).

This nanoscale size ensures a high surface-to-volume ratio, which is advantageous for interactions with biological matrices and enhances both functional and technological performance in food systems. XRD analysis (Fig. 1C) revealed a broad peak at 21.15°, attributed to the semi-crystalline structure of propolis extract and diffraction reflection from the amorphous surface^28^, along with sharper peaks at 43.97°, 64.32°, and 77.43°, which may be attributed to the JPs characteristics. It has been proposed that during the synthesis and phase separation process, one domain of the particle (likely the hydrophobic propolis-rich side) undergoes structural reorganization, forming a more ordered crystalline phase. This phenomenon can be explained by the “interface-induced crystallization” mechanism. Similar to the work of Drapak et al.^29^, who demonstrated that a propolis layer can crystallize when in contact with a structured indium monoselenide substrate, in our Janus particles, the interface between the hydrophilic (CMC) and hydrophobic domains acts as a templating surface. The interfacial energy at this boundary likely drives molecular reorganization into a more ordered crystalline lattice to minimize the free energy of the system, resulting in the observed sharp diffraction peaks. The broad propolis peak reflects a semi-crystalline arrangement without perfect crystal ordering, consistent with previous studies on propolis nanoparticles^30^. Thus, the XRD pattern clearly demonstrates the complex morphology of propolis-CMC JPs, where a semi-crystalline domain coexists with a distinct crystalline phase, confirming the true Janus nature of these particles^31^.

SEM images (Fig. 1D) exhibited a cubic morphology with rough surfaces, likely resulting from self-assembly interactions between CMC and propolis during synthesis. It is hypothesized that the high specific surface area of this structure enhances physicochemical interactions with the yogurt protein network, potentially explaining the observed improvements in the physical stability of yogurt samples enriched with propolis-CMC JPs.

**Fig. 2:**
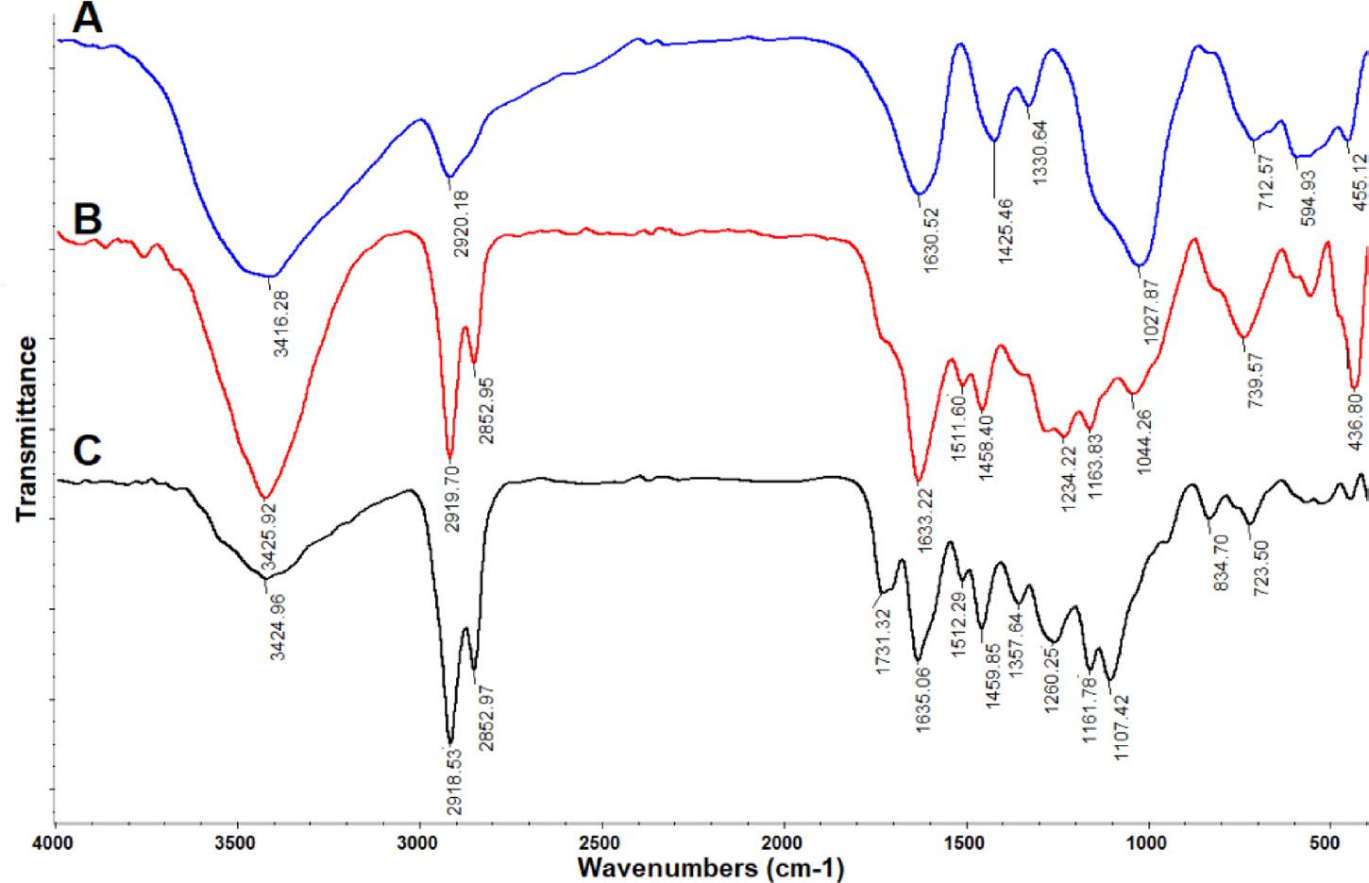
Chemical structure by FTIR (450–4000 cm^−1^): (**A**) carboxymethyl cellulose (CMC), (**B**) propolis extract, (**C**) propolis-CMC Janus nanoparticles (JPs).

FTIR spectroscopy was used to identify surface functional groups. The FTIR spectrum of CMC (Fig. 2A) revealed key structural features. A broad and intense absorption band centered at 3416 cm⁻^1^ was assigned to O–H stretching vibrations. Peaks corresponding to asymmetric C–H stretching and asymmetric –COO⁻ stretching were observed at 2920 cm⁻^1^ and 1630 cm⁻^1^, respectively. Additional bands at 1425 cm⁻^1^ and 1330 cm⁻^1^ were attributed to the symmetric stretching of carboxylic groups and the bending vibration of O–H groups, respectively. The absorption region between 1000 and 1300 cm⁻^1^, arising from C–O stretching vibrations, further confirmed the polysaccharide structure of CMC^32^. Furthermore, the FTIR spectrum of the propolis extract (Fig. 2B) exhibited absorption bands indicative of its complex phenolic composition. A broad O–H stretching band centered at 3420 cm⁻^1^ confirmed the presence of phenolic hydroxyl groups. The occurrence of long-chain alkyl compounds was supported by C–H stretching vibrations observed at 2919 cm⁻^1^ and 2852 cm⁻^1^. Characteristic bands were observed at 1633 cm⁻^1^ (attributable to C = O, C = C, and N–H vibrations), 1511 cm⁻^1^ (aromatic ring stretching), and 1458 cm⁻^1^ (C–H bending), which are associated with the skeletal aromatic rings of flavonoids and amino acids typically found in propolis. In the fingerprint region, multiple absorption bands were identified: 1336 cm⁻^1^ (C–H₂ bending of flavonoids), 1234 cm⁻^1^ (C–O stretching), and 1163 cm⁻^1^ (C–O and C–OH stretching of lipids and tertiary alcohols). Additionally, the band at 1044 cm⁻^1^ was assigned to C–C and C–OH stretching vibrations of flavonoids and secondary alcohols^33^. In the FTIR spectrum of propolis-CMC JPs (Fig. 2C), a broad band centered at 3424 cm⁻^1^ was attributed to overlapping O–H and N–H stretching vibrations. Bands at 2918 cm⁻^1^ and 2852 cm⁻^1^ corresponded to C–H stretching vibrations of methyl and methylene groups in alkyl chains. Absorption bands characteristic of propolis components were identified at 1731 cm⁻^1^ (C = O stretching), 1635 cm⁻^1^ (C = O/C = C aromatic stretching), 1512 cm⁻^1^ (C–C aromatic stretching), 1260 cm⁻^1^ (C–O stretching), 1161 cm⁻^1^ (C–OH stretching), and 1107 cm⁻^1^ (C–C stretching). Furthermore, bending vibrations of C–H and C–H₂ groups were observed at 1459 cm⁻^1^ and 1357 cm⁻^1^, respectively^18^. These functional groups are critical for antioxidant activity and potential interactions with yogurt proteins.

### Antioxidant properties

**Fig. 3:**
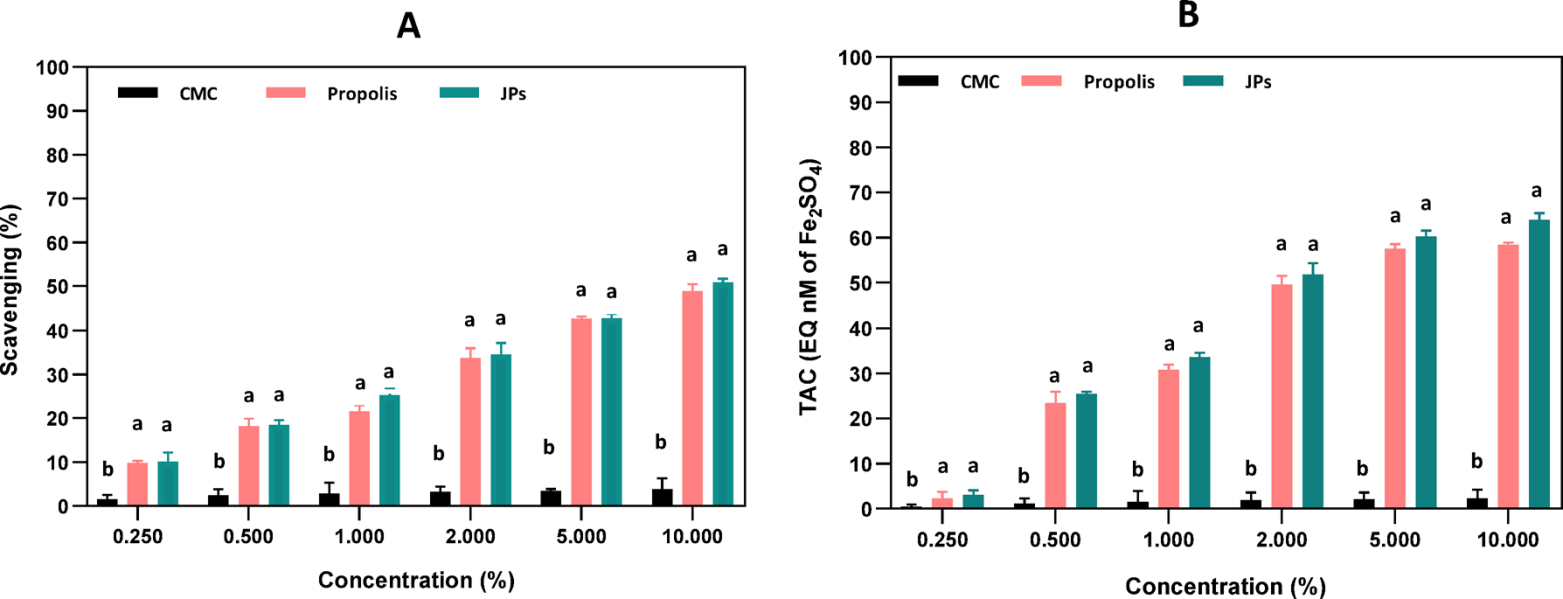
Radical scavenging activity of carboxymethyl cellulose (CMC), propolis extract, propolis-CMC Janus nanoparticles (JPs) was evaluated using DPPH assay (**A**) and the reducing antioxidant potential was assessed via FRAP assay (**B**). Different letters indicate significant differences (*p* < 0.05).

The antioxidant potential of nanoparticles is strongly influenced chemical composition, surface characteristics, concentration, and by their particle size^34^. DPPH radical scavenging and FRAP assays were employed to evaluate the antioxidant properties of CMC, propolis extract, and propolis-CMC JPs. While the DPPH assay quantifies free radical inhibition, FRAP measures the ability to reduce ferric ions (Fe^3^⁺ to Fe^2^⁺) via electron donation^35^. The results (Fig. 3), however, show that both propolis and propolis-CMC JPs have antioxidant activity, however, the antioxidant activity of propolis-CMC JPs was found to be higher than propolis extracts. Although this was not statistically significant (*p* > 0.05), it may be due to the high concentration of the active ingredients found in propolis. Propolis extracts are known to have polyphenolic compounds such as rutin, quercetin, and naringenin. The antioxidant activity of these compounds has been well documented for propolis extracts in previous studies^13^. On the contrary, CMC was less stable and have low scavenging activity. The surface functional groups like -OH, -COOH, and -CO found on the surface of the particles, as verified by FTIR (Fig. 2), are responsible for antioxidant activity, as previously reported^36^. A concentration-dependent increase in antioxidant activity was shown. As the propolis-CMC JPs concentration increased from 0.25% to 10%, both DPPH scavenging and ferric ion reduction increased significantly. It is hypothesized that the unique JPs amphiphilic nature of the nanoparticles may enhance their antioxidant properties by providing distinct domains for different interactions. One face can interact with polar radicals in aqueous environments, whereas the other can engage with nonpolar radicals, thereby increasing the overall radical-scavenging efficiency.

### Antibacterial performance

**Fig. 4:**
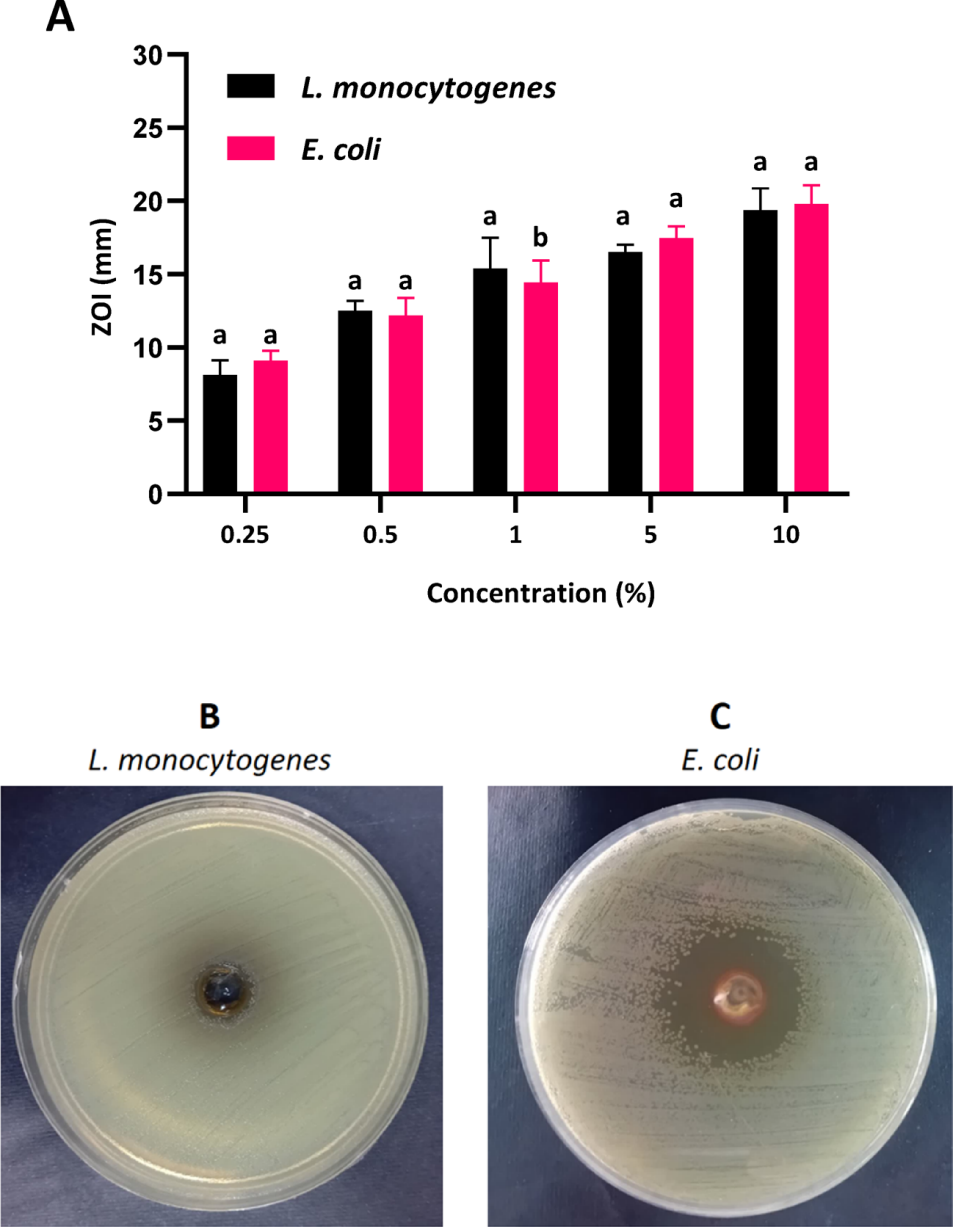
Propolis-CMC Janus nanoparticles (JPs) antimicrobial activity against *Listeria monocytogenes* and *Escherichia coli* according to agar well diffusion method as a diameter of zone of inhibition (mm) (**A**). Different letters indicate significant differences (*p* < 0.05). Zone of inhibition images of JPs at concentrations of 0.25% (**B**) and 10% against *L. monocytogenes* and *E. coli.*

The antibacterial activity of propolis-CMC JPs was evaluated against *L. monocytogenes* and *E. coli* using microdilution and agar well diffusion assays. MIC and MBC values were 1.5 mg/mL for *L. monocytogenes* and 2 mg/mL for *E. coli*. In the agar well diffusion assay, propolis-CMC JPs showed concentration-dependent antibacterial activity. At 0.25% concentration, inhibition zones (ZOI) (Fig. 4) were classified as non-sensitive (< 9 mm), whereas at 5% and 10%, propolis-CMC JPs were extremely effective (ZOI > 17 mm)^21^. This finding contrasts with that of some studies on raw propolis, which often reported higher efficacy against Gram-positive bacteria^37^. The JPs exhibited comparable antibacterial activity against both Gram-positive and Gram-negative bacteria, with no significant difference between the two groups. This finding suggests that the structure of the JPs enables effective penetration of diverse bacterial cell walls^18^. The enhanced antibacterial activity of the JPs can be attributed to their special structure. Each face possesses distinct physicochemical properties, enabling simultaneous interactions with different bacterial cell components. This structure facilitates penetration into lipid-rich bacterial membranes while maintaining interactions with aqueous environments or surface proteins, thereby increasing membrane permeability and inducing cellular damage^38^. The antibacterial effect arises from propolis active compounds, including flavonoids (kaempferol, quercetin, pinocembrin, and apigenin) and cinnamic acid derivatives, which disrupt bacterial membranes, inhibit ATPases, damage DNA/RNA, and prevent biofilm formation^12,37^. Additionally, the small size and high surface-to-volume ratio of JPs facilitate the generation of reactive oxygen species, oxidative stress, and structural alterations in bacterial cells^18^. Ma et al.^39^ showed the efficacy of their JPs, which are composed of silver nanoparticles, soybean polysaccharides, lotus leaf materials, and beeswax, as antimicrobial agents with 90% efficacy on both Gram-positive (*S. aureus*) and Gram-negative (*E. coli*) bacteria. The antimicrobial potential of JPs has been further emphasized by the research of Jia et al.^40^, wherein 0.02 mg/mL of silver-chitosan JPs inhibited the growth of both bacterial and fungal strains, including Gram-negative bacteria (*E. coli* and *Salmonella cholera*), Gram-positive bacteria (*S. aureus* and *Bacillus subtilis*), and the fungus *Botrytis cinerea*.

### Application of propolis-CMC JPs in stirred yogurt

Stirred yogurt samples were fortified with 0.5%, 1%, and 5% propolis-CMC JPs and evaluated over 21 days for their microbial, physicochemical, and sensory properties.

#### Microbial analysis

**Fig. 5:**
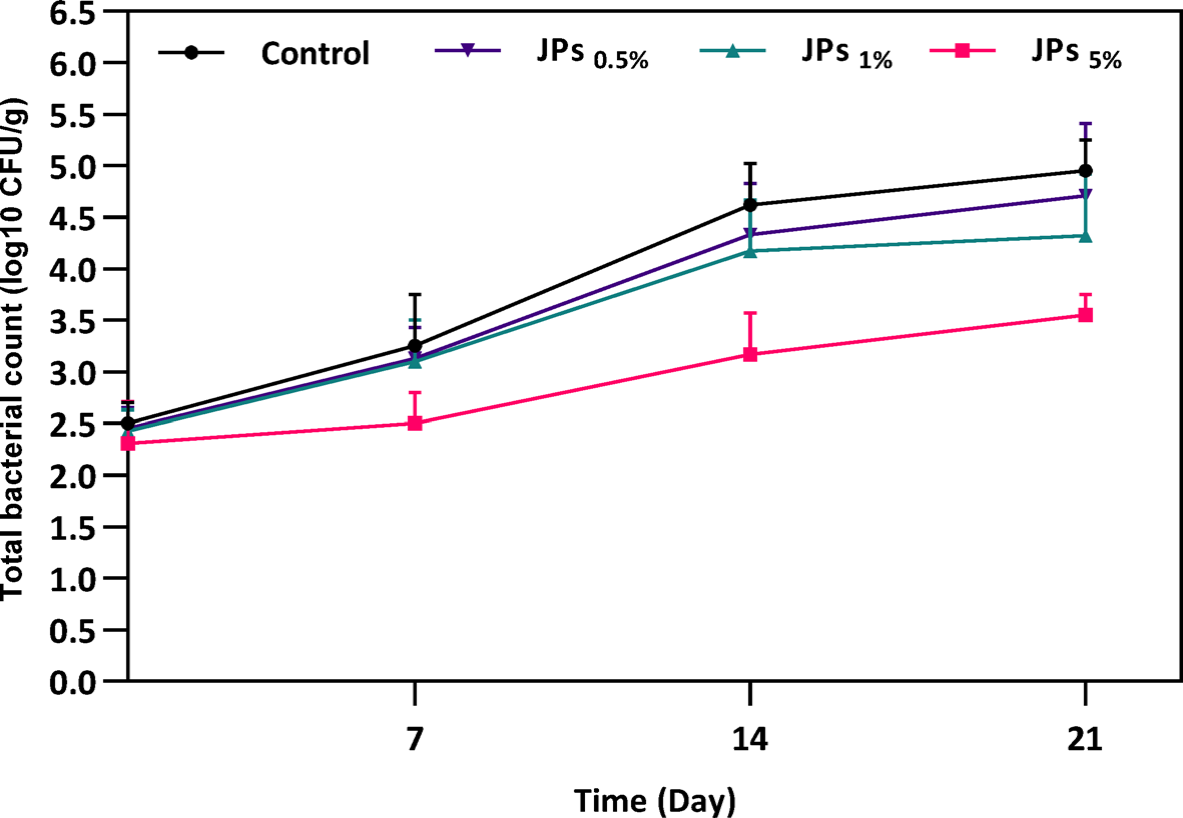
Evaluation of total bacterial count in yogurt samples containing 0.5%, 1%, and 5% (w/w) propolis-CMC Janus nanoparticles (JPs).

Figure 5 shows that the TBC in the control increased from 2.5 ± 0.2 log₁₀ CFU/g (day 0) to 4.95 ± 0.3 log₁₀ CFU/g (day 21). TBC includes both starter lactic acid bacteria and non-starter microbial flora, as enumeration was performed on a non-selective medium, reflecting the overall viable bacterial population rather than spoilage microorganisms. Propolis-CMC JPs -treated samples displayed slower microbial growth, with TBC of 4.71 ± 0.7, 4.32 ± 0.6, and 3.55 ± 0.2 log₁₀ CFU/g for 0.5%, 1%, and 5% propolis-CMC JPs, respectively. This concentration-dependent reduction is attributed to the antibacterial properties of propolis-CMC JPs, consistent with prior studies showing bioactive compounds in honey and propolis reduce bacterial proliferation in yogurt^41^. It seems that JPs asymmetry also increases the surface area available for bioactive compounds, such as flavonoids and phenolics, allowing more efficient delivery and contact with microbial cells.

#### pH and TTA

pH and TTA are interrelated yet distinct parameters that reflect the acid–base balance and metabolic state of fermented dairy systems. In yogurt systems, these two parameters are critical indicators of microbial activity, organic acid production, and overall product quality during storage. The addition of natural functional ingredients can significantly alter the acidification profile of yogurt by influencing microbial metabolism and fermentation by-products. These effects depend on the type of additive, its concentration, and the duration of storage^42^.

**Fig. 6:**
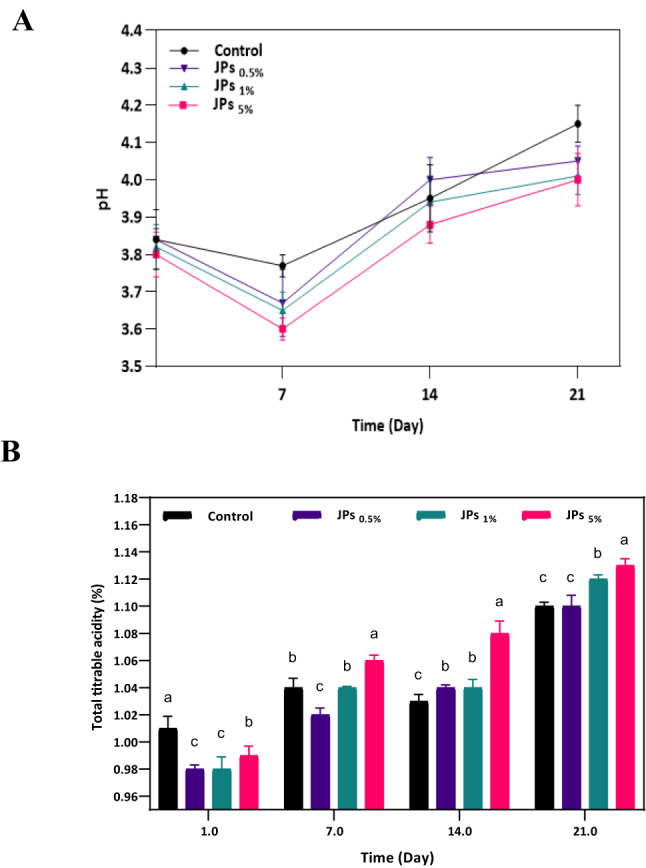
Effects of varying concentrations (0.5%, 1%, and 5% w/w) of propolis-CMC Janus nanoparticles (JPs) on pH (**A**) and total titratable acidity (**B**) in yogurt samples. Different letters indicate significant differences (*p* < 0.05).

In the present study, a clear concentration-dependent trend was observed in yogurt containing propolis-CMC JPs. Throughout the 21-day storage period, JP-supplemented samples exhibited a greater decrease in pH and a corresponding increase in TTA relative to the control (Fig. 6A and B). By day 21, the mean pH values for yogurt samples containing 0.5, 1, and 5% propolis-CMC JPs were 4.05, 4.01, and 4.00, respectively, compared with 4.15 for the control. Only the 5% propolis-CMC JPs concentration exhibited a statistically significant difference compared to the control (*p* < 0.05). This decline in pH reflects intensified lactic acid production stimulated by the bioactive components of propolis-CMC JPs, particularly phenolic and flavonoid compounds, which have been reported to modulate the metabolic activity of lactic acid bacteria. These compounds may stimulate carbohydrate utilization and organic acid production, accelerating lactic acid accumulation during storage. Similar stimulatory effects of plant-derived phenolics on LAB activity and yogurt acidification have been reported in previous studies. Interestingly, a slight pH increase on days 14 and 21 was detected, possibly associated with microbial proteolytic activity leading to amino acid deamination and amine compound synthesis, which can partially neutralize acidity. The intrinsic acidity of milk primarily arises from its citrate and phosphate buffering systems, while process-related acidity originates from lactic acid produced during fermentation by LAB. As shown in Fig. 6B, JP-enriched yogurt demonstrated a notably higher TTA than the control, indicating stronger acidification. This result aligns with the findings of Mohamed Ahmed et al.^43^, who reported that yogurt fortified with argel leaf extract exhibited a lower pH and higher TTA. They attributed this effect to the enhanced metabolic activity of LAB on the fermentable components of the extract, resulting in an increased production of organic acids. This is consistent with reports that fruit yogurt containing propolis ethanolic extract shows increased TTA and decreased pH over 28 days of storage, suggesting enhanced fermentation activity^44^. In the current study, propolis-CMC JPs likely exerted a similar stimulatory effect, fostering the metabolic efficiency of LAB and intensifying acid accumulation during storage.

#### Textural properties

**Fig. 7:**
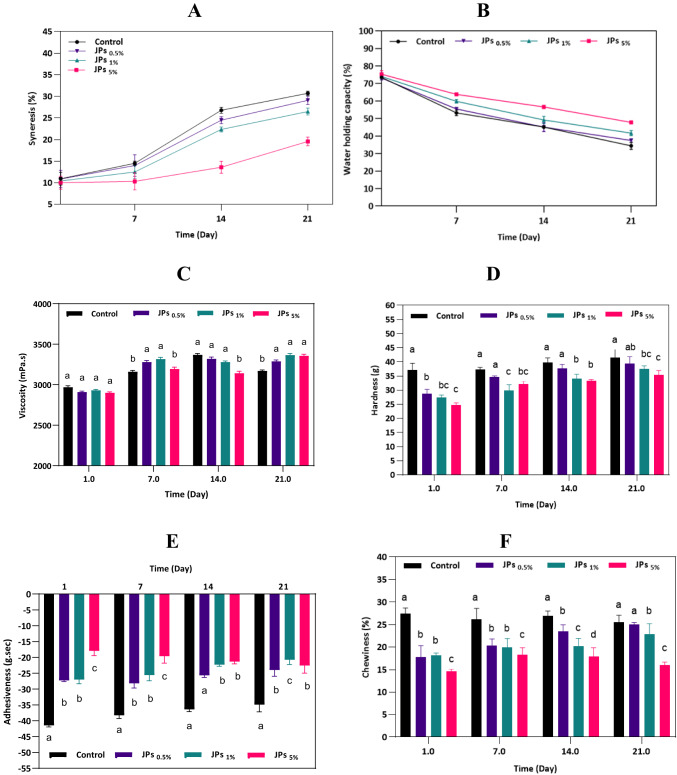
Syneresis (**A**); water absorption capacity (**B**), viscosity (**C**), hardness (**D**), adhesiveness (**E**), and chewiness (**F**) analysis of yogurt with propolis-CMC Janus nanoparticles (JPs) during 4 °C storage. Different letters indicate significant differences (*p* < 0.05).

Syneresis is a key indicator of the physical stability of yogurt, reflecting the balance between water retention and expulsion forces within the casein gel network. This provides insights into the strength and rearrangement capacity of the protein matrix^45^. As shown in Fig. 7A, syneresis gradually increased in all yogurt samples during storage. This trend can be attributed to the pH reduction that occurs over time, which causes contraction of the casein network and the subsequent expulsion of whey to the surface^46^. However, yogurt enriched with higher concentrations of propolis-CMC JPs exhibited a significant reduction in syneresis, suggesting that JPs improved the water retention capability of the gel structure. The amphiphilic nature of JPs likely facilitated better water integration within the yogurt matrix, forming a more cohesive gel that limited serum separation from the gel. This finding aligns with the concept that increased WHC can directly reduce syneresis, as JPs enhance the matrix’s ability to bind and retain moisture^47^. A similar observation was reported by Wijesekara et al.^46^, where plant-pigmented probiotic yogurt exhibited altered syneresis. This reduction in syneresis aligns with the enhanced WHC of JPs-supplemented samples, as discussed below.

WHC is another essential attribute governing yogurt stability, as it reflects its ability to retain water under gravitational or mechanical stress, such as heating, pressure, or centrifugation^48^. Incorporating natural bioactive compounds may either enhance or impair this property, depending on their affinity for water and interaction with milk proteins. In the present study, JPs-treated yogurt displayed higher WHC values than the untreated control (Fig. 7B), confirming that JPs improved the internal structural stability and moisture-binding efficiency of the product.

Hardness, a critical rheological and sensory parameter, strongly influences consumer preferences and marketability. As depicted in Fig. 7D, the incorporation of propolis-CMC JPs led to a slight reduction in hardness compared with the control. This reduction is likely due to the hydrophilic characteristics of propolis-CMC JPs, which increase water content and gel softness. The interaction between propolis-CMC JPs and casein micelles enhanced water retention while simultaneously reducing gel compactness, resulting in a softer texture. Although the enrichment of propolis-CMC JPs increased the apparent viscosity, the simultaneous enhancement of water retention slightly weakened the gel-network. This led to a reduction in hardness, as viscosity reflects the flow behavior of the system, whereas hardness reflects the firmness of the gel under compression. Similar results were observed by Wu et al.^49^, who found that the addition of rice bran weakened yogurt structure by producing a brittle and porous gel. Additionally, fat globules are known to influence rheological behavior, loss or destabilization of these particles can compromise yogurt quality. In contrast, fortification with natural stabilizers, such as non-enzymatic gums or orange peel fibers, has been shown to protect fat globules, thereby maintaining gel cohesion and textural integrity^50^.

Adhesiveness represents the work required to overcome the attractive forces between yogurt and a contact surface. As seen in Fig. 7E, adhesiveness increased gradually in all samples during storage, consistent with the findings of Jooyandeh et al.^25^. Nonetheless, JP-enriched yogurts demonstrated lower adhesiveness values than the control, possibly due to the formation of a smoother and more uniform protein–polyphenol network that reduced stickiness. Chewiness, which determines the ability of yogurt to maintain its structural form and mouthfeel during mastication, is an important rheological parameter affecting consumer sensory experience. Over the storage period, yogurts containing propolis-CMC JPs showed improved chewiness compared to their initial states (Fig. 7F). This enhancement suggests that the inclusion of propolis-CMC JPs contributed to the development of a more elastic and cohesive gel matrix, reflecting the potential of these nanoparticles to positively influence the textural and mechanical properties of yogurt.

#### Rheological properties

As shown in Fig. 7C, no significant difference in viscosity was observed between the control and samples containing 0.5% and 1% propolis-CMC JPs during the first 14 d of storage. However, by day 21, all JP-enriched yogurts exhibited significantly higher viscosity (*p* < 0.05) than the control, with the highest value recorded for the yogurt containing 5% propolis-CMC JPs. This improvement may be attributed to progressive acidification, leading to the formation of dense protein–polyphenol complexes that reinforce the structural integrity of the gel^51^. Moreover, natural bioactive compounds can modify the yogurt microstructure by forming micropores and fine capillary channels, resulting in a compact and uniform network^3,52^. The inclusion of gel-forming or texturizing functional ingredients helps preserve essential texture attributes, such as hardness, cohesiveness, and viscosity^53^.

#### Antioxidant activity

**Fig. 8:**
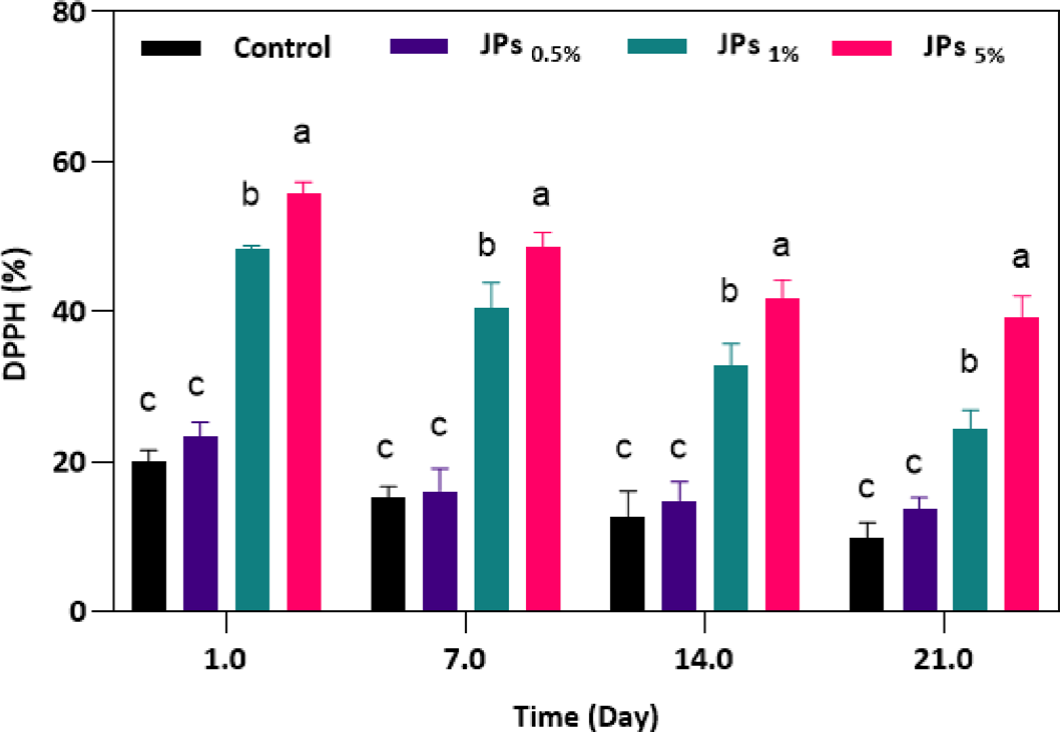
DPPH assay measured the antioxidant radical-scavenging activity of yogurts enriched with propolis-CMC Janus nanoparticles (JPs). Different letters indicate significant differences (*p* < 0.05).

Incorporating natural additives into yogurt can significantly enhance its antioxidant potential, primarily due to the enrichment with bioactive compounds such as phenolic acids, flavonoids, polysaccharides, and anthocyanins^54^. The antioxidant capacity of the yogurt samples was assessed using the DPPH radical scavenging assay (Fig. 8). Control-stirred yogurt exhibited the lowest percentage of radical inhibition, while the incorporation of propolis-CMC JPs significantly (*p* < 0.05) improved the scavenging activity in a concentration-dependent manner. This trend is consistent with the earlier findings of this study regarding the intrinsic antioxidant behavior of propolis-CMC JPs (Fig. 3). The enhanced activity can be attributed to the distinctive amphiphilic structure of the propolis-CMC JPs, which allows better dispersion of antioxidant molecules within both hydrophilic and hydrophobic regions of the yogurt matrix. Such structural asymmetry facilitates more effective radical neutralization in heterogeneous food systems. Furthermore, the surface phenolic groups of the JPs may act as active scavenging sites capable of donating hydrogen to stabilize DPPH radicals. Comparable outcomes have been documented in previous studies. Tami et al.^55^ reported that buffalo milk yogurt fortified with grape seed extract exhibited superior antioxidant potential compared to the control, with activity increasing proportionally with extract concentration due to the presence of catechin, epicatechin, and proanthocyanidin oligomers. It seems that the addition of propolis-CMC JPs into yogurt not only increased its antioxidant activity but also provided a more stable distribution of antioxidant compounds, supporting the potential of Janus-based materials as functional ingredients for fortified dairy systems.

#### Sensory evaluation and color parameters

**Fig. 9:**
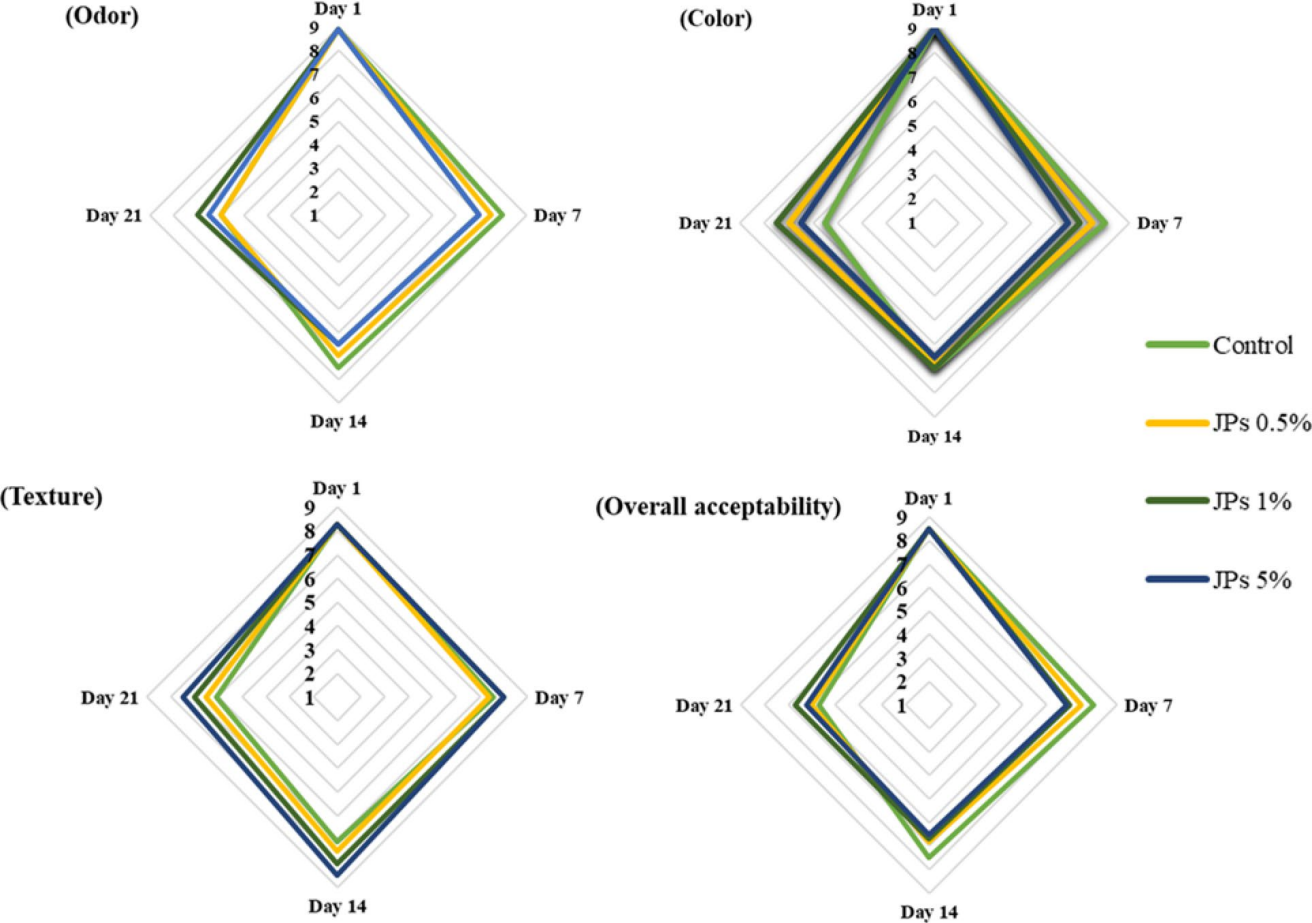
Sensory changes on different days in control and yogurt treated with propolis-CMC Janus nanoparticles (JPs) at concentrations of 0.5%, 1%, and 5% (w/w) during 4 °C storage.

As shown in Fig. 9, the addition of propolis-CMC JPs significantly improved the overall acceptability of stirred yogurt samples. Among the formulations, yogurts containing 1% propolis-CMC JPs achieved the highest sensory scores for color and odor, whereas the sample enriched with 5% propolis-CMC JPs received the highest score for texture. These results indicate that moderate JP enrichment can optimize sensory balance, while higher concentrations may enhance structural properties due to the texturizing effect of propolis-CMC JPs within the protein matrix.

Objective color measurements were expressed as L* (lightness), a* (red-green component), and b* (yellow-blue component) values. Throughout the storage period, all yogurt samples showed a gradual increase in b* values (Table 1), indicating an increase in yellowness over time. This change can be attributed to the instability of casein micelles and protein–pigment interactions as pH decreased during storage. Compared with the control sample, the addition of propolis-CMC JPs resulted in a decline in L* values, reflecting reduced brightness, alongside increases in both a* and b* parameters. This concentration-dependent color shift corresponded to greater redness and yellowness with increasing propolis-CMC JPs content. Specifically, a* values shifted from negative to positive, and b* values increased significantly as the propolis-CMC JPs concentration increased, imparting a more pronounced yellowish hue to the yogurt. These color modifications may arise from the presence of natural pigments and phenolic compounds in propolis, as well as their interactions with the protein and polysaccharide components of the yogurt matrix. The amphiphilic structure of propolis-CMC JPs enhances pigment dispersion and stability within both aqueous and lipid phases, leading to uniform coloration and minimized phase separation. No significant differences in ΔE values were observed between yogurt samples on day 7. However, on day 21, all propolis-CMC JPs enriched yogurt samples exhibited significantly lower ΔE compared to the control, indicating a notable protective effect of propolis-CMC JPs against color changes during storage.

**Table 1:** The changes in the color (L*, a*, and b*) and total color difference (ΔE) of yogurt enriched with different concentrations propolis-CMC Janus nanoparticles (JPs) over a 21-day storage.

	Treatments				
	Days	Control	JPs 0.5%	JPs1%	JPs5%
L*	1	95.4 ± 0.4a	92.1 ± 0.1b	90.2 ± 0.2bc	88.8 ± 0.6c
	7	87.6 ± 0.2a	84.0 ± 0.6b	83.5 ± 0.4b	81.4 ± 0.5c
	14	84.1 ± 0.5a	82.5 ± 0.5b	80.9 ± 0.2b	77.6 ± 0.6c
	21	80.1 ± 0.2a	78.5 ± 0.5b	75.6 ± 0.7c	74.5 ± 0.6c
a*	1	− 1.7 ± 0.2b	− 0.1 ± 0.3c	− 1.1 ± 0.3b	− 2.3 ± 0.4a
	7	1.2 ± 0.4c	1.9 ± 0.7ab	1.6 ± 0.8bc	2.2 ± 0.6a
	14	0.8 ± 0.6c	1.0 ± 0.2c	1.8 ± 0.1b	5.8 ± 0.7a
	21	5.9 ± 0.5a	2.4 ± 0.3c	3.3 ± 0.2b	3.9 ± 0.4b
b*	1	1.1 ± 0.2bc	0.9 ± 0.2c	1.8 ± 0.6b	3.2 ± 0.7a
	7	2.8 ± 0.1c	3.6 ± 0.4b	4.1 ± 0.4a	3.5 ± 0.7b
	14	7.7 ± 0.1c	8.1 ± 0.4b	8.5 ± 0.6b	9.4 ± 0.4a
	21	7.8 ± 0.4b	7.4 ± 0.1b	9.5 ± 0.2a	9.9 ± 0.5a
∆E	1	0.0 ± 0.0a	0.0 ± 0.0a	0.0 ± 0.0a	0.0 ± 0.0a
	7	8.4 ± 0.3a	8.7 ± 0.6a	7.5 ± 0.8a	8.6 ± 0.3a
	14	13.3 ± 0.4b	12.0 ± 0.4bc	11.8 ± 0.3c	15.1 ± 0.5a
	21	18.3 ± 0.5a	15.2 ± 0.7c	17.0 ± 0.5b	16.9 ± 0.4b

Within each row, values with different letters are significantly different (*p* < 0.05).

Natural acidification process during yogurt fermentation, typically characterized by a drop in pH and an increase in titratable acidity, can further influence color perception^56^. Comparable trends have been reported in yogurts fortified with phenolic-rich extracts such as olive leaf or grape skin powders, where polyphenol–protein complex formation alters spectral properties and reduces lightness^57^. Taken together, the results indicate that propolis-CMC JPs incorporation improves the visual appeal of stirred yogurt and stabilizes its color parameters over storage, reinforcing their suitability as multifunctional color-modifying and antioxidant ingredients for functional dairy applications.

### Conclusions

This study successfully demonstrated that propolis-CMC JPs act as a novel, multifunctional ingredient capable of enhancing the overall quality and functionality of stirred yogurt. Structural characterization confirmed the nanoscale size (13.40 nm ± 3.56 μm) and amphiphilic configuration of propolis-CMC JPs, enabling their simultaneous role as natural emulsifiers, antimicrobial agents, and antioxidants within the yogurt matrix. Incorporation of propolis-CMC JPs, particularly at a 5% concentration, significantly reduced syneresis, improved viscosity, and strengthened the gel network, while also enhancing antioxidant capacity and inhibiting microbial growth during storage. Sensory evaluation revealed that yogurt containing 5% propolis-CMC JPs achieved the highest scores for texture, whereas the 1% propolis-CMC JPs formulation was preferred in terms of color and odor, reflecting an optimal balance between visual, textural, and aromatic attributes. The amphiphilic nature of propolis-CMC JPs provides a uniform dispersion of phenolic compounds, leading to enhanced oxidative stability and consistent sensory performance throughout storage. This study presents a sustainable and consumer-friendly approach for developing next-generation functional dairy products. Future work should focus on long-term storage stability and in vivo safety assessment of propolis-CMC JPs to support their industrial application.

## Data Availability

The datasets used and/or analyzed during the current study available from the corresponding author on reasonable request.

## References

[1] Guirguis, E. H. & Meleka, M. B. Effect of thyme (*Thymus vulgaris* L.) essential oil on the quality parameters of stirred yoghurt. *J. Med. Sci. Res.* **3**, 265 (2020).

[2] Ibrahim, E. A., El-Sayed, S. M., Murad, S. A., Abdallah, W. E. & El-Sayed, H. S. Evaluation of the antioxidant and antimicrobial activities of fucoxanthin from Dilophys fasciola and as a food additive in stirred yoghurt. *S. Afr. J. Sci.* **119**, 3–4 (2023).

[3] Zhu, H. et al. Physicochemical, sensory, and antioxidant characteristics of stirred-type yogurt enriched with *Lentinula edodes* stipe powder. *Food Sci. Nutr.* **11**, 6231–6240 (2023). 37823167 10.1002/fsn3.3563 PMC10563725

[4] Allam, A. et al. Plain set and stirred yogurt with different additives: Implementation of food safety system as quality checkpoints. *PeerJ* **11**, e14648 (2023). 36726723 10.7717/peerj.14648 PMC9885861

[5] Shanuke, D. S., Edirisinghe, E. M. R. K. B., Marapana, R. A. U. J. & Hettiarachi, S. Development of omega-3 fortified stirred yoghurt with enhanced sensory and oxidative qualities through the addition of fish oil nanoemulsion and fruits. *Int. J. Food Sci. Technol.* **60**, vvae034 (2025).

[6] Chu, X. et al. A review on properties and antibacterial applications of polymer-functionalized carbon dots. *J. Mater. Sci.* **57**, 12752–12781 (2022).

[7] Tabari Shahandasht, N., Bolandi, M., Rahmati, M. & Jafarisani, M. Enhancing stirred yogurt quality with hyaluronic acid-rich rooster comb extract: Effects on texture and shelf life. *Food Sci. Nutr.* **13**, e4666 (2025). 39803235 10.1002/fsn3.4666 PMC11717033

[8] Li, X. et al. Preparation and application of Janus nanoparticles: Recent development and prospects. *Coord. Chem. Rev.* **454**, 214–318 (2022).

[9] Liu, Z. et al. Janus particles: A review of their applications in food and medicine. *Crit. Rev. Food Sci. Nutr.*10.1080/10408398.2022.2067831 (2022) (**In press**). 35475710 10.1080/10408398.2022.2067831

[10] Wei, D., Ge, L., Lu, S., Li, J. & Guo, R. Janus particles templated by Janus emulsions and application as a Pickering emulsifier. *Langmuir* **33**, 5819–5828 (2017). 28541052 10.1021/acs.langmuir.7b00939

[11] Li, D., Jiang, J. & Cai, C. Palladium nanoparticles anchored on amphiphilic Janus-type cellulose nanocrystals for Pickering interfacial catalysis. *Chem. Commun.* **56**, 9396–9399 (2020). 10.1039/d0cc03892j 32676633

[12] Segueni, N. et al. Review on propolis applications in food preservation and active packaging. *Plants* **12**, 1654 (2023). 37111877 10.3390/plants12081654 PMC10142627

[13] Siripatrawan, U. & Vitchayakitti, W. Improving functional properties of chitosan films as active food packaging by incorporating with propolis. *Food Hydrocoll.* **61**, 695–702 (2016).

[14] Yong, H. & Liu, J. Active packaging films and edible coatings based on polyphenol-rich propolis extract: A review. *Compr. Rev. Food Sci. Food Saf.* **20**, 2106–2145 (2021). 33486883 10.1111/1541-4337.12697

[15] Zabaiou, N. et al. Biological properties of propolis extracts: Something new from an ancient product. *Chem. Phys. Lipids* **207**, 214–222 (2017). 28411017 10.1016/j.chemphyslip.2017.04.005

[16] Chen, L., Ao, F., Ge, X. & Shen, W. Food-grade pickering emulsions: Preparation, stabilization and applications. *Molecules* **25**, 3202 (2020). 32674301 10.3390/molecules25143202 PMC7397194

[17] Chang, X., Feng, W., He, L., Chen, X. & Liang, L. Fabrication and characterisation of whey protein isolate–propolis–alginate complex particles for stabilising α-tocopherol-contained emulsions. *Int. Dairy J.* **109**, 104756 (2020).

[18] Razavi, R., Tajik, H., Molaei, R., McClements, D. J. & Moradi, M. Janus nanoparticles synthesized from hydrophobic carbon dots and carboxymethyl cellulose: Novel antimicrobial additives for fresh food applications. *Food Biosci.* **62**, 105171 (2024).

[19] Nikfarjam, N., Razavi, R., Moradi, M. & Molaei, R. Green synthesis of carbon dots from onion juice and ex-situ embedding for antimicrobial/ultraviolet protective nanocellulose films. *Food Saf. Packag.* **1**, 24–32 (2025).

[20] Razavi, R. et al. Biosynthesis of metallic nanoparticles using mulberry fruit (Morus alba L.) extract for the preparation of antimicrobial nanocellulose film. *Appl. Nanosci.* **10**, 465–476 (2020).

[21] Ghorbani, M., Tajik, H., Moradi, M., Molaei, R. & Alizadeh, A. One-pot microbial approach to synthesize carbon dots from baker’s yeast-derived compounds for the preparation of antimicrobial membrane. *J. Environ. Chem. Eng.* **10**, 107525 (2022).

[22] Gonçalves, R. F. S., Rodrigues, R., Vicente, A. A. & Pinheiro, A. C. Incorporation of solid lipid nanoparticles into stirred yogurt: Effects in physicochemical and rheological properties during shelf-life. *Nanomaterials* **13**, 93 (2023). 10.3390/nano13010093 PMC9823338 36616003

[23] Qiu, L., Zhang, M., Mujumdar, A. S. & Chang, L. Effect of edible rose (*Rosa rugosa* cv. Plena) flower extract addition on the physicochemical, rheological, functional and sensory properties of set-type yogurt. *Food Biosci.* **43**, 101249 (2021).

[24] Ghaderi-Ghahfarokhi, M., Shakarami, M. & Zarei, M. Alfalfa sprout flour: A promising ingredient to improve probiotic survival and antioxidant activity in mint-flavored stirred yogurt. *Appl. Food Res.* **4**, 100555 (2024).

[25] Jooyandeh, H., Momenzadeh, S., Alizadeh Behbahani, B. & Barzegar, H. Effect of *Malva neglecta* and lactulose on survival of *Lactobacillus fermentum* and textural properties of synbiotic stirred yogurt. *J. Food Sci. Technol.* **60**, 1136–1143 (2023). 36908339 10.1007/s13197-023-05667-6 PMC9998791

[26] Pham, Q.-H. et al. Yogurts fortified with postbiotic powders derived from *Lactobacillus acidophilus* LA5: Physicochemical, rheological, antioxidant, and sensory properties. *LWT* **213**, 117043 (2024).

[27] Shori, A. B., Albalawi, A., Al Zahrani, A. J., Al-sulbi, O. S. & Baba, A. S. Microbial analysis, antioxidant activity, and sensory properties of yoghurt with different starter cultures during storage. *Int. Dairy J.* **126**, 105267 (2022).

[28] Drapak, S. I., Bakhtinov, A. P., Gavrylyuk, S. V., Kovalyuk, Z. D. & Lytvyn, O. S. The formation of organic (propolis films)/inorganic (layered crystals) interfaces for optoelectronic applications. *Superlattices Microstruct.* **44**, 563–570 (2008).

[29] Drapak, S. I., Bakhtinov, A. P., Gavrilyuk, S. V., Prylutskyy, Y. I. & Kovalyuk, Z. D. X-ray diffraction investigation of the structure of propolis films. *Phys. Solid State* **48**, 1602–1604 (2006).

[30] Shirvani, A., Goli, S. A. H., Varshosaz, J., Salvia-Trujillo, L. & Martín-Belloso, O. Fabrication of edible solid lipid nanoparticle from beeswax/propolis wax by spontaneous emulsification: Optimization, characterization and stability. *Food Chem.* **387**, 132934 (2022). 35421652 10.1016/j.foodchem.2022.132934

[31] Hessberger, T. et al. Co-flow microfluidic synthesis of liquid crystalline actuating Janus particles. *J. Mater. Chem. C* **4**, 8778–8786 (2016).

[32] Ezati, P., Rhim, J. W., Moradi, M., Tajik, H. & Molaei, R. CMC and CNF-based alizarin incorporated reversible pH-responsive color indicator films. *Carbohydr. Polym.* **246**, 116614 (2020). 32747254 10.1016/j.carbpol.2020.116614

[33] Woźniak, M., Kwaśniewska-Sip, P., Krueger, M., Roszyk, E. & Ratajczak, I. Chemical, biological and mechanical characterization of wood treated with propolis extract and Silicon compounds. *Forests* **11**, 907 (2020).

[34] Daré, R. G. & Lautenschlager, S. O. S. Nanoparticles with antioxidant activity. *Antioxidants* **14**, 221 (2025). 40002407 10.3390/antiox14020221 PMC11852090

[35] Cömert, E. D. & Gökmen, V. Evolution of food antioxidants as a core topic of food science for a century. *Food Res. Int.* **105**, 76–93 (2018). 29433271 10.1016/j.foodres.2017.10.056

[36] Rahman, M. S. et al. Recent developments of carboxymethyl cellulose. *Polymers* **13**, 35 (2021). 10.3390/polym13081345 PMC8074295 33924089

[37] Almuhayawi, M. S. Propolis as a novel antibacterial agent. *Saudi J. Biol. Sci.* **27**, 3079–3086 (2020). 33100868 10.1016/j.sjbs.2020.09.016 PMC7569119

[38] Alizadeh, N., Moradi, M., Molaei, R. & Razavi, R. Bacterial nanocellulose films functionalized with Janus nanoparticles: Preparation and application in chicken meat preservation and safety. *Sci. Rep.* **7**, 1–56 (2026). 10.1038/s41598-026-39029-x PMC12932641 41652087

[39] Ma, Z. et al. Lotus leaf inspired sustainable and multifunctional Janus film for food packaging. *Chem. Eng. J.* **457**, 141279 (2023).

[40] Jia, R., Jiang, H., Jin, M., Wang, X. & Huang, J. Silver/chitosan-based Janus particles: Synthesis, characterization, and assessment of antimicrobial activity in vivo and vitro. *Food Res. Int.* **78**, 433–441 (2015). 28433312 10.1016/j.foodres.2015.08.035

[41] Abe, M. M., Branciforti, M. C. & Brienzo, M. Biodegradation of hemicellulose-cellulose-starch-based bioplastics and microbial polyesters. *Recycling* **6**, 22 (2021).

[42] Han, Y., Jin, X., Zhang, Z. & Sun, Q. Characterization of pectin with different structural features and its effects on yogurt quality. *LWT* **229**, 118236 (2025).

[43] Mohamed Ahmed, I. A. et al. Physicochemical quality attributes and antioxidant properties of set-type yogurt fortified with argel (*Solenostemma argel* Hayne) leaf extract. *LWT* **137**, 110389 (2021).

[44] Güney, F. & Ertürk, Ö. Determination of the effects of propolis ethanolic extract on some properties of fruit yoghurt during storage. *Mustafa Kemal Üniversitesi Tarım Bilim. Derg.* **25**, 145–152 (2020).

[45] Arab, M. et al. A comprehensive review on yogurt syneresis: effect of processing conditions and added additives. *J. Food Sci. Technol.* **60**, 1656–1665 (2023). 37187980 10.1007/s13197-022-05403-6 PMC10169984

[46] Wijesekara, A., Weerasingha, V., Jayarathna, S. & Priyashantha, H. Quality parameters of natural phenolics and its impact on physicochemical, microbiological, and sensory quality attributes of probiotic stirred yogurt during the storage. *Food Chem. X* **14**, 100332 (2022). 35634218 10.1016/j.fochx.2022.100332 PMC9130075

[47] Wong, S.-S., Wicklund, R., Bridges, J., Whaley, J. & Koh, Y. B. Starch swelling behavior and texture development in stirred yogurt. *Food Hydrocoll.* **98**, 105274 (2020).

[48] Demirci, T. *et al.* Influence of hot and cold break tomato powders on survival of probiotic L. paracasei subsp. paracasei F19, texture profile and antioxidative activity in set-type yoghurts.* LWT* **118**, 108855 (2020).

[49] Wu, T., Deng, C., Luo, S., Liu, C. & Hu, X. Effect of rice bran on properties of yogurt: Comparison between addition of bran before fermentation and after fermentation. *Food Hydrocoll.* **135**, 108122 (2023).

[50] Mary, P. R., Mutturi, S. & Kapoor, M. Non-enzymatically hydrolyzed guar gum and orange peel fibre together stabilize the low-fat, set-type yogurt: A techno-functional study. *Food Hydrocoll.* **122**, 107100 (2022).

[51] Dönmez, Ö., Mogol, B. A. & Gökmen, V. Syneresis and rheological behaviors of set yogurt containing green tea and green coffee powders. *J. Dairy Sci.* **100**, 901–907 (2017). 28012628 10.3168/jds.2016-11262

[52] Rashwan, A. K. et al. Chemical composition, quality attributes and antioxidant activity of stirred-type yogurt enriched with *Melastoma dodecandrum* Lour fruit powder. *Food Funct.* **13**, 1579–1592 (2022). 35073395 10.1039/d1fo03448k

[53] Pachekrepapol, U., Kokhuenkhan, Y. & Ongsawat, J. Formulation of yogurt-like product from coconut milk and evaluation of physicochemical, rheological, and sensory properties. *Int. J. Gastron. Food Sci.* **25**, 100393 (2021).

[54] Shori, A. B. Storage quality and antioxidant properties of yogurt fortified with polyphenol extract from nutmeg, black pepper, and white pepper. *Electron. J. Biotechnol.* **57**, 24–30 (2022).

[55] Tami, S. H., Aly, E., Darwish, A. A. & Mohamed, E. S. Buffalo stirred yoghurt fortified with grape seed extract: New insights into its functional properties. *Food Biosci.* **47**, 101752 (2022).

[56] Akarca, G. & Denizkara, A. J. Changes of quality in yoghurt produced under magnetic field effect during fermentation and storage processes. *Int. Dairy J.* **150**, 105841 (2024).

[57] Tarchi, I., Koubaa, M., Ozogul, F., Bouaziz, M. & Aït-Kaddour, A. Influence of olive leaf extract on the physicochemical properties of yogurts made from cow, sheep, and goat milk. *Food Biosci.* **63**, 105728 (2025).

